# Serum choline is associated with hepatocellular carcinoma survival: a prospective cohort study

**DOI:** 10.1186/s12986-020-00445-z

**Published:** 2020-03-30

**Authors:** Zhao-Yan Liu, Dinuerguli Yishake, Ai-Ping Fang, Dao-Ming Zhang, Gong-Cheng Liao, Xu-Ying Tan, Yao-Jun Zhang, Hui-Lian Zhu

**Affiliations:** 1 Guangdong Provincial Key Laboratory of Food, Nutrition and Health, School of Public Health, Sun Yat-sen University, Guangzhou, 510080 People’s Republic of China; 2 Department of Hepatobiliary Surgery, Sun Yat-sen University Cancer Center, Guangzhou, 510060 People’s Republic of China

**Keywords:** Choline, Betaine, Hepatocellular carcinoma, Survival

## Abstract

**Background:**

Higher choline and betaine levels have been linked to lower risk of liver cancer, whereas existing data in relation to hepatocellular carcinoma (HCC) prognosis are scarce. Our objective was to examine the associations of the serum choline and betaine with HCC survival.

**Methods:**

866 newly diagnosed HCC patients were enrolled in the Guangdong Liver Cancer Cohort. Serum choline and betaine were assessed using high-performance liquid chromatography with online electro-spray ionization tandem mass spectrometry. Liver cancer-specific survival (LCSS) and overall survival (OS) were calculated. Cox proportional hazards models were used to estimate the hazard ratios (HRs) and 95% confidence intervals (CIs).

**Results:**

Serum choline levels were associated with better LCSS (T3 vs. T1: HR = 0.69, 95% CI: 0.51–0.94; *P*_-trend_ < 0.05) and OS (T3 vs. T1: HR = 0.73, 95% CI: 0.54–0.99; *P*_-trend_ < 0.05). The associations were significantly modified by C-reactive protein (CRP) levels but not by other selected prognostic factors including sex, age, etc. The favorable associations between serum choline and LCSS and OS were only existed among patients with CRP ≥3.0 mg/L. No significant associations were found between serum betaine levels and either LCSS or OS.

**Conclusions:**

This study revealed that higher serum choline levels were associated with better HCC survival, especially in HCC patients with systemic inflammation status. No significant associations were found between serum betaine and HCC survival. Our findings suggest the benefits of choline on HCC survival.

**Trial registration:**

The Guangdong Liver Cancer Cohort was registered at clinicaltrials.gov as NCT03297255.

## Background

Hepatocellular carcinoma (HCC) is one of the most common malignancy, and is a leading cause of cancer-related morbidity and mortality worldwide [1]. Multiple well-established ways aimed for curative treatment of HCC patients. However, the prognosis is still poor with five-year survival in the range of 5–30% [1, 2]. To improve long-term survival and diminish suffering of HCC patients, many convenient modifiable ways have been proposed to guide patient care, among which favorable dietary nutrients and nutritional status [3, 4] remain as major challenges.

Choline is an essential nutrient present in all cells, and plays a wide range of physiological functions in the body [5]. For example, choline is important to maintain the structural integrity of cellular membranes by serving as a component of phosphatidylcholine, which comprises almost half of the cellular membranes [6]. And it is needed to form acetylcholine, an important signaling neurotransmitter [6]. In addition, choline occupies critical roles in maintaining normal liver function. Particularly, choline is a major source of methyl groups via oxidation to betaine, their functions involving the re-methylation of homocysteine to methionine are critical for DNA and histone methylation homeostasis, and therefore have been reported to be associated with cancer risk [2] (including liver cancer) and survival [7–10]. Experimental studies have reported that when deprived of choline, varying degrees of liver damage and liver diseases developed, including elevated transaminases [11], affected lipid metabolism and transport [12], fatty liver [13], liver cirrhosis [13] and even liver cancer [14], whereas choline or betaine supplementation ameliorated liver damage [15, 16], and choline supplementation increased global DNA methylation and DNA methyltransferase expression in HepG2 cells [17], which implied that choline or betaine might not only be associated with hepatocarcinogenesis, but also with liver cancer survival. To date, although scarcely, three case control studies [18–20] have evaluated the relationship between choline and betaine and human liver cancer risk, in which consistently favorable effects of choline were found. Of note, previous studies reported seriously insufficient dietary choline intake in Chinese population [20, 21], and approximately 50% of the total HCC cases occurred in China [19]. No existing studies have reported the associations between choline and betaine and liver cancer survival, it would be a very meaningful topic to investigate the influence of choline and betaine status on HCC prognosis in Chinese population.

In this study, we aimed to examine serum choline and betaine in relation to HCC survival in the Guangdong Liver Cancer Cohort (GLCC), to testify the hypothesis that choline and betaine status would be associated with HCC survival.

## Methods

### Study population

GLCC is an ongoing prospective cohort study conducted at Sun Yat-sen University Cancer Center (SYSUCC), the detailed protocols have been described previously [22, 23]. The present analyses included adult HCC patients enrolled between September 2013 and February 2017 if they met all the following criteria: 1) diagnosed within 1 month, 2) had no history of other carcinoma or any anticancer therapy, 3) had donated available fasting blood samples. HCC was diagnosed by either imaging or histopathology based on National Comprehensive Cancer Network Clinical Practice Guidelines in Oncology: Hepatobiliary Cancers [24].

### Serum choline and betaine measurement

Fasting blood samples were collected as soon as possible once recruited before any anticancer therapies. Serum concentrations of choline and betaine were assessed by high-performance liquid chromatography with online electro-spray ionization tandem mass spectrometry to measure the concentrations of choline, and betaine. Detailed protocol of choline measurement has been described previously [19]. Detection of serum betaine was similar to choline, except for the internal standard was d9-betaine (obtained from Sigma-Aldrich, St. Louis, USA). The coefficients of variation for the between-run assays were 4.91 and 6.21% for choline and betaine, respectively.

### Survival data collection

The date of recruitment was used as the start date of follow-up for GLCC patients, and the last outcome follow-up of the present study was conducted on September 26, 2017. We assessed liver cancer-specific survival (LCSS) and overall survival (OS), and deaths from liver cancer or any cause were assigned as outcome event of LCSS or OS, respectively. The ascertainment process of deaths has been described previously [22, 23].

### Other covariates

A face-to-face structured questionnaire was used to collect sociodemographic and lifestyle data by well-trained research staff at baseline. Current smokers or current alcohol drinkers were patients who smoked more than one cigarette per day or drank alcohol more than once per week continuously during past six months, and patients who had quit smoking or drinking at diagnosis were former smokers or former drinkers. Body mass index (BMI) was calculated based on height (m) and weight (kg) at diagnosis. In addition, education level, age at diagnosis, sex, residence and family history of primary liver cancer were also collected.

We retrospectively extracted examination, diagnostic and treatment data from the SYSUCC electronic medical records. Laboratory data at diagnosis included albumin (ALB), total bilirubin (TBIL), alkaline phosphatase (ALP), γ-glutaryl-transferase (GGT), aspartate aminotransferase (AST), alanine aminotransferase (ALT), prothrombin time (PT), alpha fetoprotein (AFP), C-reactive protein (CRP), hepatitis B surface antigen (HBsAg) and hepatitis C virus antibody (Anti-HCV). A liver damage level was computed based on six liver function tests (ALB, TBIL, ALP, GGT, AST and ALT), cutoffs were described previously [22], the level was presented as 1 (no liver injury), 2 (possible minor injury), and **≥** 3 (possible injury) in this study. Characteristics of HCC tumor and the non-tumoral liver were reviewed through imaging or pathology data at diagnosis, then Child-Pugh class [25] and Barcelona Clinic Liver Cancer (BCLC) stage [25] were determined. Treatments mainly included liver resection, intervention, radiofrequency ablation and others. In addition, presence of chronic diseases at diagnosis which may affect the prognosis of HCC were also collected, including hypertension, diabetes mellitus, fatty liver and cirrhosis.

### Statistical analysis

The statistical analyses were computed in SPSS version 20.0 for WINDOWS (SPSS Inc., Chicago, IL, USA). All *P*-values were based on two-sided tests, and *P* < 0.05 was considered statistically significant.

The serum levels of choline and betaine at diagnosis were mostly rescaled to sex-specific tertiles (T) for analysis, except for the stratified analyses. Differences in basic characteristics across sex-specific tertiles of serum choline and betaine were compared by one-way analysis of variance (ANOVA), Kruskal-Wallis tests or Pearson’s Chi-Squared tests, where appropriate. Multivariate Cox proportional hazards models were used to examine the relationship between serum levels of choline and betaine and HCC survival outcomes. Hazard ratios (HRs) and 95% confidence interval (CI) were calculated with the first tertiles as the reference. Before performing multivariate analysis, covariates were preliminarily screened using the nonparametric log-rank tests (*P*-values for log-rank test are presented in Additional file 1: Supplementary Table 1), and those with *P*-values > 0.05 were firstly excluded. Then the multicollinearity was evaluated using the variation inflation factor and tolerance. Presence of fatty liver, presence of cirrhosis and Child-Pugh class were further excluded, due to the multicollinearity with the baseline liver damage level or BCLC stage. Particularly, age at diagnosis and sex (log-rank test *P*-values > 0.05) were retained while residence (log-rank test *P*-value < 0.05) was excluded, since age and sex are well-established HCC prognostic factors. Finally, in model 1, nonclinical covariates such as age at diagnosis, sex, education level, BMI and smoking status were selected to adjust. In model 2, significant clinical covariates were further adjusted, including AFP level (≥400 or < 400 ng/L), CRP level (≥3.0 or < 3 mg/L), baseline liver damage level, BCLC stage and treatments. Linear trends (*P*_-trend_) were evaluated by entering sex-specific tertiles of serum choline and betaine as continuous variables in the corresponding models.

Stratified analyses were conducted to evaluate whether the relationships between serum choline and betaine and HCC survival would be modified by potential prognosis factors for HCC, including sex, age at diagnosis, BMI, smoking status, alcohol drinking status, serum folate level, AFP level, CRP level, baseline liver damage level, presence of fatty liver, presence of cirrhosis, BCLC stage and treatments. Interactions were estimated by including the multiplicative interaction terms in the multivariate models.

## Results

### Patient characteristics

Basic characteristics of the 866 HCC patients by sex are presented in Additional file 2 (Supplementary Table 2), and by sex-specific tertiles of serum choline and betaine are shown in Table 1. Of the 866 patients, there were 767 (88.6%) men. The mean (±S.D.) age was 52.3 ± 11.8 years. The median (IQR) levels of serum choline and betaine were 11.72 (9.52–14.92) μmol/L and 64.48 (52.44–77.65) μmol/L, respectively. Compared to subjects in the bottom sex-specific tertiles of serum choline, those in the top tertiles were older, less likely to be current smokers, more likely to be former smokers and were less been diagnosed as BCLC C stage. Compared to patients in the bottom sex-specific tertiles of serum betaine, those in the top tertiles were more living in rural, more infected by HBV or HCV and less suffered from fatty liver.

**Table 1:** Patient characteristics at diagnosis by sex-specific tertiles of serum choline/betaine levels

	Serum choline				Serum betaine			
	T1	T2	T3	***P***-value ^**b**^	T1	T2	T3	***P***-value ^**b**^
**Age at diagnosis**^**a**^**, years**	50.2 ± 11.9	52.8 ± 11.5	53.8 ± 11.7	0.00	51.6 ± 11.7	52.1 ± 12.2	51.6 ± 11.7	0.37
**Men,*n*(%)**	257 (88.6)	257 (88.9)	256 (89.2)	0.98	256 (88.9)	258 (89.0)	256 (88.9)	1.00
**BMI at diagnosis**^**a**^**, kg/m**^**2**^	22.5 ± 3.3	22.8 ± 3.2	22.7 ± 3.3	0.34	22.5 ± 3.0	22.9 ± 3.6	22.5 ± 3.0	0.68
**Education level,*n*(%)**				0.37				0.45
Primary school or below	56 (19.4)	63 (21.9)	52 (18.1)		62 (21.7)	48 (16.6)	61 (21.2)	
Secondary & High school	168 (58.3)	177 (61.5)	183 (63.8)		175 (61.2)	184 (63.7)	169 (58.7)	
College or above	64 (22.2)	48 (16.7)	52 (18.1)		49 (17.1)	57 (19.7)	58 (20.1)	
**Residence,*n*(%)**				0.60				0.03
Urban	195 (67.2)	205 (70.9)	195 (67.9)		215 (74.7)	192 (66.2)	188 (65.3)	
Rural	95 (32.8)	84 (29.1)	92 (32.1)		73 (25.3)	98 (33.8)	100 (34.7)	
**Smoking status,*n*(%)**				0.00				0.06
Current	101 (34.8)	90 (31.1)	74 (25.8)		106 (36.8)	81 (27.9)	78 (27.1)	
former	55 (19.0)	86 (29.8)	100 (34.8)		71 (24.7)	80 (27.6)	90 (31.2)	
Never	134 (46.2)	113 (39.1)	113 (39.4)		111 (38.5)	129 (44.5)	120 (41.7)	
**Alcohol drinking status,*n*(%)**				0.57				0.73
Current	83 (28.6)	75 (26.0)	79 (27.5)		75 (26.0)	86 (29.7)	76 (26.4)	
former	39 (13.4)	37 (12.8)	48 (16.7)		41 (14.2)	37 (12.8)	46 (16.0)	
Never	168 (57.9)	177 (61.2)	160 (55.7)		172 (59.7)	167 (57.6)	166 (57.6)	
**With family history of PLC,*n*(%)**	46 (15.9)	33 (11.4)	33 (11.5)	0.19	37 (12.8)	39 (13.4)	36 (12.5)	0.94
**HBV or HCV infected,*n*(%)**	269 (92.8)	263 (91.0)	257 (89.5)	0.40	254 (88.2)	261 (90.0)	274 (95.1)	0.01
**AFP ≥ 400 ng/L,*n*(%)**	112 (38.9)	130 (45.0)	116 (40.4)	0.30	124 (43.2)	124 (42.9)	110 (38.2)	0.39
**CRP ≥ 3.0 mg/L,*n*(%)**	148 (51.6)	139 (48.3)	149 (52.1)	0.61	138 (48.1)	137 (47.6)	161 (56.3)	0.06
**Presence of chronic diseases,*n*(%)**								
Hypertension	28 (9.7)	44 (15.2)	39 (13.6)	0.12	34 (11.7)	33 (11.5)	111 (12.8)	0.31
Diabetes mellitus	19 (6.6)	21 (7.3)	29 (10.1)	0.25	30 (10.4)	22 (7.6)	17 (5.9)	0.13
Fatty liver	44 (15.2)	50 (17.3)	49 (17.1)	0.75	64 (22.2)	47 (16.2)	32 (11.1)	0.00
Cirrhosis	176 (60.7)	186 (64.4)	182 (63.4)	0.64	175 (60.8)	176 (60.7)	193 (67.0)	0.20
**Baseline liver damage level,*n*(%)**				0.87				0.16
1	58 (20.0)	62 (21.5)	52 (18.1)		60 (20.8)	58 (20.0)	54 (18.8)	
2	109 (37.6)	109 (37.7)	116 (40.4)		95 (33.0)	123 (42.4)	116 (40.3)	
3	123 (42.4)	118 (40.8)	119 (41.5)		133 (46.2)	109 (37.6)	118 (41.0)	
**Child-Pugh class,*n*(%)**				0.45				0.16
A	283 (97.6)	286 (99.0)	282 (98.3)		286 (99.3)	285 (98.3)	280 (97.2)	
B	7 (2.4)	3 (1.0)	5 (1.7)		2 (0.7)	5 (1.7)	8 (2.8)	
**BCLC stage,*n*(%)**				0.01				0.95
0	23 (7.9)	31 (10.7)	28 (9.8)		25 (8.7)	28 (9.7)	29 (10.1)	
A	85 (29.3)	80 (27.7)	101 (35.2)		87 (30.2)	95 (32.8)	84 (29.2)	
B	20 (6.9)	41 (14.2)	34 (11.8)		33 (11.5)	32 (11.0)	30 (10.4)	
C	162 (55.9)	137 (47.4)	124 (43.2)		143 (49.7)	135 (46.6)	145 (50.3)	
**Treatment,*n*(%)**				0.61				0.18
Liver resection	132 (45.5)	124 (42.9)	127 (44.3)		133 (46.2)	134 (46.2)	116 (40.3)	
Radiofrequency ablation	31 (10.7)	28 (9.7)	26 (9.1)		21 (7.3)	29 (10.0)	35 (12.2)	
Intervention	109 (37.6)	127 (43.9)	121 (42.2)		121 (42.0)	109 (37.6)	127 (44.1)	
Others	18 (6.2)	10 (3.5)	13 (4.5)		13 (4.5)	18 (6.2)	10 (3.5)	
**Serum choline (μmol/L)**				< 0.001				< 0.001
Median	8.76	11.77	18.14		10.48	11.83	12.97	
Interquartile range	8.13–9.53	11.03–12.54	14.94–20.36		8.93–12.89	9.62–14.07	10.37–17.11	
**Serum betaine (**μmol**/L)**				< 0.001				< 0.001
Median	59.93	66.08	73.65		48.19	64.49	83.72	
Interquartile range	47.13–70.24	54.44–74.30	59.08–85.95		42.13–53.35	60.91–68.60	77.65–94.46	
**Serum folate (μmol/L)**				< 0.001				0.03
Median	7.13	6.97	8.29		6.75	7.52	7	
Interquartile range	5.30–8.54	4.70–8.88	5.90–10.37		5.05–8.87	5.47–9.71	5.20–9.44)	

^a^ values are expressed as mean ± S.D.

^b^*P*-values were calculated by one-way analysis of variance (ANOVA), Kruskal-Wallis tests and Pearson’s Chi-Squared tests, where appropriate

### Associations between serum choline and betaine with HCC survival outcomes

The median survival of HCC patients was 467 days (IQR: 227–835 days). During the follow-up, 291 (33.6%) deaths were documented, 270 (92.8%) of which were attributable to liver cancer. Both liver cancer-specific and overall mortality decreased with sex-specific tertiles of serum choline levels after adjusting for nonclinical factors in model 1. The associations became even stronger after further adjustment for traditional HCC prognostic factors in model 2, HRs (95% CIs) of liver cancer-specific and overall mortality (T3 vs T1) in model 2 were 0.69 (0.51–0.94) and 0.73 (0.54–0.99), respectively. Both *P*_-trend_ values were < 0.05. However, no significant associations were found between serum betaine levels and either LCSS or OS in the fully-adjusted model 2 (all *P*_-trend_ values were > 0.05). Data are presented in Table 2.

**Table 2:** Survival outcomes by sex-specific tertiles of serum choline and betaine levels

	T1	T2	T3	***P***_**-trend**_
**Liver cancer-specific survival**				
Serum choline				
Deaths / Total cases	87 / 290	84 / 289	99 / 287	
Person-days at risk	122,905	143,328	202,856	
Model 1-adjusted HR (95% CI) ^a^	1.00	0.83 (0.61–1.13)	0.73 (0.55–0.99)	0.04
Model 2-adjusted HR (95% CI) ^b^	1.00	0.84 (0.62–1.15)	0.69 (0.51–0.94)	0.02
Serum betaine				
Deaths / Total cases	90 / 288	94 / 290	86 / 288	
Person-days at risk	149,722	156,715	162,652	
Model 1-adjusted HR (95% CI) ^a^	1.00	0.94 (0.70–1.26)	0.85 (0.63–1.15)	0.29
Model 2-adjusted HR (95% CI) ^b^	1.00	0.97 (0.72–1.31)	0.84 (0.62–1.13)	0.25
**Overall survival**				
Serum choline				
Deaths / Total cases	90 / 290	91 / 289	110 / 287	
Person-days at risk	122,905	143,328	202,856	
Model 1-adjusted HR (95% CI) ^a^	1.00	0.85 (0.63–1.14)	0.78 (0.58–1.05)	0.11
Model 2-adjusted HR (95% CI) ^b^	1.00	0.85 (0.62–1.15)	0.73 (0.54–0.99)	0.04
Serum betaine				
Deaths / Total cases	97 / 288	99 / 290	95 / 288	
Person-days at risk	149,722	156,715	162,652	
Model 1-adjusted HR (95% CI) ^a^	1.00	0.93 (0.70–1.24)	0.88 (0.66–1.17)	0.39
Model 2-adjusted HR (95% CI) ^b^	1.00	0.95 (0.72–1.27)	0.84 (0.63–1.13)	0.25

^a^ Covariates adjusted in Model 1: age, sex, BMI, education level, smoking status

^b^ Covariates adjusted in Model 2: covariates adjusted in Model 1, additionally adjusted for baseline liver damage level, BCLC stage, treatment, AFP level and CRP level

### Stratified analyses

Stratified analyses of serum choline and betaine levels by selected factors are shown in Tables 3 and 4. No statistically significant multiplicative effect modifications were found across most of the aforementioned strata factors, either for LCSS or OS (all *P*_-interaction_ > 0.05). However, we observed significant multiplicative effect modifications by CRP level for serum choline. Compared to the first tertile of serum choline, patients in the third tertile had better survival in CRP ≥3.0 mg/L strata (HRs (95% CIs) of LCSS and OS (T3 vs T1) were 0.60 (0.41–0.88) and 0.63 (0.44–0.91), *P*_-interaction_ = 0.010 and 0.005, respectively.) but not in CRP < 3.0 mg/L strata.

**Table 3:** HR (95% CI) of stratified analysis across tertiles of serum choline levels

	Liver cancer-specific survival					Overall survival				
	T1	T2	T3	*P*_-trend_	*P*_-interaction_	T1	T2	T3	*P*_-trend_	*P*_-interaction_
**Sex**^**a, b**^					0.511					0.781
Men	1.00	0.77 (0.56–1.07)	0.66 (0.48–0.92)	0.013		1.00	0.80 (0.58–1.10)	0.71 (0.52–0.98)	0.037	
Women	1.00	1.21 (0.41–3.52)	1.59 (0.57–4.44)	0.360		1.00	1.15 (0.42–3.14)	1.33 (0.50–3.54)	0.567	
**Age**^**a, b**^**, years**					0.838					0.801
< 60	1.00	1.03 (0.72–1.48)	0.69 (0.48–0.99)	0.031		1.00	1.07 (0.75–1.52)	0.74 (0.52–1.06)	0.070	
≥ 60	1.00	0.80 (0.42–1.51)	0.63 (0.34–1.17)	0.142		1.00	0.83 (0.45–1.53)	0.65 (0.36–1.18)	0.155	
**BMI**^**a, b**^**, kg/m**^**2**^					0.357					0.436
< 25.0	1.00	0.90 (0.64–1.27)	0.74 (0.53–1.04)	0.079		1.00	0.88 (0.62–1.23)	0.77 (0.55–1.07)	0.117	
≥ 25.0	1.00	1.15 (0.54–2.44)	0.47 (0.20–1.10)	0.118		1.00	1.03 (0.50–2.12)	0.61 (0.28–1.32)	0.185	
**Smoking status**^**a, b**^					0.820					0.756
Smoker	1.00	0.58 (0.39–0.85)	0.57 (0.39–0.83)	0.010		1.00	0.61 (0.42–0.89)	0.61 (0.42–0.88)	0.013	
Non-smoker	1.00	1.17 (0.69–1.98)	0.85 (0.50–1.42)	0.481		1.00	1.19 (0.71–2.01)	0.91 (0.55–1.51)	0.669	
**Drinking status**^**a, b**^					0.388					0.399
Drinker	1.00	0.67 (0.41–1.07)	0.78 (0.50–1.22)	0.342		1.00	0.71 (0.44–1.13)	0.75 (0.48–1.16)	0.237	
Non-Drinker	1.00	1.23 (0.82–1.86)	0.72 (0.47–1.12)	0.077		1.00	1.16 (0.77–1.74)	0.74 (0.49–1.13)	0.135	
**AFP level**^**a, b**^**, ng/mL**					0.160					0.566
≥ 400	1.00	0.85 (0.56–1.30)	0.80 (0.52–1.22)	0.300		1.00	0.86 (0.57–1.29)	0.83 (0.56–1.25)	0.385	
< 400	1.00	0.87 (0.55–1.37)	0.68 (0.43–1.06)	0.085		1.00	0.91 (0.58–1.42)	0.73 (0.48–1.13)	0.155	
**CRP level**^**a, b**^**, mg/L**					0.010					0.005
≥ 3.0	1.00	0.91 (0.64–1.29)	0.60 (0.41–0.88)	0.008		1.00	0.92 (0.65–1.29)	0.63 (0.44–0.91)	0.013	
< 3.0	1.00	1.34 (0.70–2.56)	1.37 (0.74–2.51)	0.353		1.00	1.34 (0.71–2.54)	1.47 (0.81–2.67)	0.224	
**Serum folate level**^**a, b**^					0.782					0.946
High	1.00	1.07 (0.67–1.70)	0.79 (0.50–1.26)	0.279		1.00	1.17 (0.74–1.84)	0.91 (0.58–1.43)	0.583	
Low	1.00	0.96 (0.62–1.47)	0.77 (0.50–1.17)	0.207		1.00	0.94 (0.62–1.42)	0.76 (0.50–1.14)	0.170	
**Liver damage level**^**a, b**^					0.520					0.602
1	1.00	1.39 (0.34–5.74)	1.54 (0.40–6.00)	0.547		1.00	1.85 (0.52–6.62)	1.16 (0.34–3.97)	0.935	
2	1.00	1.06 (0.63–1.78)	0.58 (0.33–1.01)	0.045		1.00	1.06 (0.63–1.78)	0.65 (0.38–1.13)	0.114	
≥ 3	1.00	0.82 (0.55–1.22)	0.72 (0.48–1.08)	0.115		1.00	0.86 (0.58–1.26)	0.78 (0.53–1.14)	0.206	
**Presence of fatty liver**^**a, b**^					0.385					0.842
Yes	1.00	1.08 (0.38–3.07)	0.94 (0.32–2.78)	0.902		1.00	1.05 (0.40–2.76)	1.17 (0.45–3.03)	0.741	
No	1.00	0.94 (0.68–1.30)	0.69 (0.50–0.97)	0.026		1.00	0.96 (0.70–1.32)	0.72 (0.52–0.99)	0.035	
**Presence of cirrhosis**^**a, b**^					0.518					0.170
Yes	1.00	0.93 (0.60–1.43)	0.83 (0.54–1.27)	0.380		1.00	0.93 (0.61–1.42)	0.90 (0.60–1.35)	0.602	
No	1.00	0.98 (0.63–1.52)	0.63 (0.39–1.00)	0.048		1.00	1.01 (0.66–1.56)	0.62 (0.39–0.98)	0.039	
**BCLC stage**^**a, b**^					0.774					0.502
0/A	1.00	0.80 (0.34–1.90)	0.72 (0.32–1.62)	0.440		1.00	0.91 (0.40–2.11)	0.78 (0.35–1.73)	0.515	
B/C	1.00	0.89 (0.64–1.23)	0.64 (0.46–0.90)	0.008		1.00	0.88 (0.63–1.21)	0.67 (0.48–0.93)	0.014	
**Treatment**^**a, b**^					0.333					0.480
Liver resection	1.00	1.29 (0.69–2.41)	0.81 (0.42–1.56)	0.536		1.00	1.16 (0.63–2.14)	0.87 (0.46–1.62)	0.664	
Intervention	1.00	0.75 (0.51–1.10)	0.60 (0.41–0.86)	0.006		1.00	0.80 (0.55–1.17)	0.64 (0.45–0.92)	0.015	
Others	1.00	1.26 (0.52–3.07)	0.95 (0.37–2.42)	0.908		1.00	1.21 (0.49–2.94)	1.00 (0.40–2.50)	0.985	

^a^ Covariates adjusted for age, sex, BMI, education level, smoking status, baseline liver damage level, BCLC stage, treatment, AFP level and CRP level

^b^ Stratified factors were not included in the corresponding models

**Table 4:** HR (95% CI) of stratified analysis across tertiles of serum betaine levels

	Liver cancer-specific survival					Overall survival				
	T1	T2	T3	*P*_-trend_	*P*_-interaction_	T1	T2	T3	*P*_-trend_	*P*_-interaction_
**Sex**^**a, b**^					0.229					0.328
Men	1.00	0.95 (0.70–1.29)	0.73 (0.53–0.99)	0.046		1.00	0.94 (0.70–1.27)	0.76 (0.56–1.03)	0.078	
Women	1.00	0.91 (0.30–2.74)	1.30 (0.45–3.74)	0.590		1.00	0.83 (0.31–2.24)	1.36 (0.53–3.47)	0.504	
**Age**^**a, b**^**, years**					0.627					0.721
< 60	1.00	1.01 (0.72–1.43)	0.88 (0.62–1.25)	0.460		1.00	1.01 (0.73–1.41)	0.89 (0.64–1.25)	0.505	
≥ 60	1.00	1.17 (0.65–2.13)	0.86 (0.47–1.58)	0.586		1.00	1.19 (0.67–2.14)	0.90 (0.50–1.62)	0.678	
**BMI**^**a, b**^**, kg/m**^**2**^					0.702					0.574
< 25.0	1.00	1.01 (0.73–1.39)	0.85 (0.61–1.18)	0.331		1.00	0.96 (0.70–1.32)	0.84 (0.62–1.15)	0.276	
≥ 25.0	1.00	2.06 (0.89–4.75)	1.78 (0.76–4.15)	0.218		1.00	1.90 (0.87–4.15)	1.37 (0.61–3.07)	0.526	
**Smoking status**^**a, b**^					0.097					0.050
Smoker	1.00	1.16 (0.81–1.68)	0.76 (0.52–1.11)	0.153		1.00	1.13 (0.79–1.61)	0.79 (0.55–1.13)	0.187	
Non-smoker	1.00	0.90 (0.54–1.50)	1.04 (0.64–1.69)	0.884		1.00	0.89 (0.54–1.46)	1.05 (0.65–1.69)	0.837	
**Drinking status**^**a, b**^					0.176					0.227
Drinker	1.00	0.96 (0.61–1.51)	0.98 (0.62–1.54)	0.932		1.00	0.97 (0.62–1.52)	0.98 (0.63–1.52)	0.935	
Non-Drinker	1.00	0.99 (0.67–1.46)	0.78 (0.52–1.17)	0.226		1.00	0.97 (0.66–1.41)	0.77 (0.52–1.13)	0.187	
**AFP level**^**a, b**^**, ng/mL**					0.527					0.194
≥ 400	1.00	1.03 (0.69–1.54)	0.96 (0.63–1.45)	0.853		1.00	0.94 (0.64–1.38)	0.93 (0.63–1.38)	0.703	
< 400	1.00	1.25 (0.81–1.95)	0.87 (0.55–1.37)	0.609		1.00	1.28 (0.83–1.99)	0.98 (0.63–1.52)	0.832	
**CRP level**^**a, b**^**, mg/L**					0.329					0.379
≥ 3.0	1.00	0.87 (0.61–1.23)	0.87 (0.61–1.24)	0.446		1.00	0.83 (0.59–1.17)	0.89 (0.63–1.24)	0.504	
< 3.0	1.00	1.34 (0.78–2.31)	0.70 (0.38–1.31)	0.287		1.00	1.39 (0.82–2.35)	0.81 (0.45–1.46)	0.525	
**Serum folate level**^**a, b**^					0.250					0.387
High	1.00	1.27 (0.82–1.96)	0.86 (0.54–1.37)	0.545		1.00	1.17 (0.77–1.78)	0.91 (0.59–1.41)	0.674	
Low	1.00	1.03 (0.68–1.54)	0.92 (0.62–1.38)	0.695		1.00	1.01 (0.68–1.51)	0.93 (0.63–1.37)	0.700	
**Liver damage level**^**a, b**^					0.145					0.313
1	1.00	1.66 (0.50–5.49)	0.63 (0.14–2.86)	0.536		1.00	1.19 (0.40–3.55)	0.60 (0.17–2.14)	0.396	
2	1.00	1.46 (0.87–2.45)	1.11 (0.66–1.89)	0.716		1.00	1.47 (0.88–2.47)	1.24 (0.74–2.09)	0.430	
≥ 3	1.00	0.83 (0.56–1.24)	0.84 (0.57–1.24)	0.398		1.00	0.84 (0.57–1.23)	0.84 (0.58–1.22)	0.361	
**Presence of fatty liver**^**a, b**^					0.664					0.333
Yes	1.00	1.39 (0.46–4.19)	1.49 (0.53–4.20)	0.461		1.00	1.56 (0.57–4.24)	1.76 (0.69–4.51)	0.245	
No	1.00	1.04 (0.77–1.42)	0.81 (0.59–1.11)	0.194		1.00	1.03 (0.76–1.39)	0.83 (0.61–1.12)	0.225	
**Presence of cirrhosis**^**a, b**^					0.136					0.069
Yes	1.00	1.28 (0.85–1.95)	0.89 (0.59–1.35)	0.552		1.00	1.27 (0.85–1.91)	0.95 (0.64–1.40)	0.732	
No	1.00	1.04 (0.67–1.60)	0.76 (0.48–1.19)	0.164		1.00	0.87 (0.57–1.35)	0.71 (0.46–1.10)	0.118	
**BCLC stage**^**a, b**^					0.653					0.632
0/A	1.00	1.51 (0.69–3.27)	1.06 (0.46–2.46)	0.900		1.00	1.63 (0.76–3.50)	1.11 (0.52–2.40)	0.822	
B/C	1.00	0.94 (0.66–1.33)	0.84 (0.59–1.19)	0.325		1.00	0.91 (0.65–1.28)	0.87 (0.62–1.21)	0.405	
**Treatment**^**a, b**^					0.292					0.305
Liver resection	1.00	1.24 (0.67–2.30)	1.02 (0.53–1.98)	0.945		1.00	1.14 (0.64–2.03)	0.88 (0.48–1.64)	0.720	
Intervention	1.00	0.82 (0.57–1.17)	0.67 (0.46–0.97)	0.032		1.00	0.82 (0.58–1.17)	0.72 (0.50–1.02)	0.065	
Others	1.00	1.41 (0.57–3.48)	0.84 (0.32–2.20)	0.668		1.00	1.47 (0.60–3.64)	1.03 (0.41–2.59)	0.974	

^a^ Covariates adjusted for age, sex, BMI, education level, smoking status, baseline liver damage level, BCLC stage, treatment, AFP level and CRP level

^b^ Stratified factors were not included in the corresponding models

## Discussion

To our best knowledge, this is the first study to examine serum choline and betaine in relation to HCC survival in a large cohort. We observed that higher serum choline levels at diagnosis were associated with better HCC survival, especially in those with systemic inflammation status (CRP level ≥ 3.0 mg/L), whereas serum betaine had no statistically significant associations with HCC survival outcomes.

Given its critically protective role for liver and other essential physiological functions in the body, it is not surprising that serum choline demonstrated favorable associations with HCC survival. First, choline is an essential nutrient for maintaining liver function. Liver is among the first organs to accumulate choline absorbed from the intestine and responsible for most of choline metabolism [26]. Experimental studies have proved that choline deficiency might cause varying degrees of liver diseases ranging from dyslipidemia to liver cancer [26], whereas choline or betaine supplementation could ameliorate liver damage [15, 16]. Second, though choline is essential for maintaining normal liver function, its functions are more than this. It is indispensable for normal function of all cells, including the structural integrity of cell membranes and neurotransmission [27]. Thus, adequate choline can improve general health of HCC patients so as to further improve HCC survival outcomes. Third, choline is a major source of methyl group via oxidation to betaine, their important roles in methylation reactions are critical for DNA and histone methylation homeostasis. Jiang et al. [17] used the human hepatic HepG2 cell to examine the effect of choline supplementation on DNA methylation, they found that choline supplementation increased both global DNA methylation and DNA methyltransferase expression, suggesting that choline could improve prognosis of HCC. However, in the present study, the favorable associations were only observed between serum choline and HCC survival outcomes, and no statistically significant associations between serum betaine and HCC survival outcomes were found. These contradictory results implied that the protective effect of choline on HCC survival may not through the methylation metabolic pathway via oxidation to betaine. As a methyl donor, betaine participates in the re-methylation of homocysteine to methionine via the enzyme betaine homocysteine methyltransferase (BHMT) [26]. However, this key enzyme is greatly reduced in HCC patients [28], and downregulation of BHMT in HCC associates with poor prognosis [29]. Besides, after treating the Hepa 1–6 (derived cells from a mouse HCC model) and E47/C34 cell lines (clones of the HepG2 cell line) with exogenous S-adenosylmethionine (SAM) and betaine, results showed that SAM decreased the number of Hepa 1–6 and E47/C34 cells, and increased the number of dead cells in vitro, while betaine had no significant effect either on the number of surviving cells or dead cells [30]. Lastly, in the stratified analysis, we observed significant multiplicative effect modifications by CRP level, the favorable association between serum choline and HCC survival was only found in CRP ≥3.0 mg/L strata, but not in CRP < 3.0 mg/L strata. As a marker of systemic inflammation status, CRP has been identified as a useful prognostic factor for HCC [31]. Our results implied that elevating serum choline may have more beneficial effects on HCC patients with systemic inflammation status.

Several strengths of this study should be noted. This is the first large prospective cohort study to investigate the associations of serum choline with HCC survival in a Chinese population, whose dietary choline intake were seriously insufficient [20, 21], thus the value of this study is significant and our findings may have impact for the design of future clinical trials. We only enrolled newly diagnosed HCC patients and collected blood samples within 1 months of diagnosis, to eliminate potential confounding by receiving any anticancer treatments. Furthermore, we collected very detailed information of the HCC patients, including both general and clinical prognostic factors.

However, some potential limitations should also be considered. We failed to collect the information of dietary habits changes and disease progression during the follow-up, and we had only one measurement of serum choline and betaine at diagnosis, due to the high mortality and poor prognosis of HCC, another time-consuming face-to-face interview and blood collection was extremely difficult, thus we cannot know the circulating choline and betaine changes after diagnosis. In addition, it should be noted that our study is a preliminary epidemiological research, although to some extent, our finding had opened up new prospect in understanding the beneficial role of choline on HCC survival, we cannot explain the exact mechanisms restricted by the observational study design, further investigations such as randomized clinical trials are warranted to confirm these findings.

## Conclusions

In conclusion, the key findings from this large prospective cohort study revealed that higher serum choline levels at diagnosis were associated with better HCC survival outcomes, independently from representative nonclinical and clinical prognostic factors, especially in HCC patients with systemic inflammation status. Further investigations such as randomized clinical trials are warranted to confirm these findings.

## Supplementary information

**Additional file 1 Table S1.** Screening procedure of variables by nonparametric log-rank test.

**Additional file 2 Table S2.** Baseline characteristics of female and male HCC patients.

## Data Availability

The datasets generated and/or analyzed during the current study are not publicly available due to privacy protection of the participants but are available from the corresponding author on reasonable request.

## Abbreviations

HCC: Hepatocellular carcinoma
GLCC: Guangdong Liver Cancer Cohort
LCSS: Liver cancer-specific survival
OS: Overall survival
HRs: Hazard ratios
CIs: Confidence intervals
SYSUCC: Sun Yat-sen University Cancer Center
PLC: Primary liver cancer
T1: First tertile
T2: Second tertile
T3: Third tertile
BMI: Body mass index
HBV: Hepatitis B virus
HCV: Hepatitis C virus
AFP: Alpha fetoprotein
CRP: C-reactive protein
BCLC: Barcelona Clinic Liver Cancer
ALB: Albumin
TBIL: Total bilirubin
ALP: Alkaline phosphatase
GGT: γ-glutaryl-transferase
AST: Aspartate aminotransferase
ALT: Alanine aminotransferase
PT: Prothrombin time
SAM: S-adenosylmethionine
BHMT: Betaine homocysteine methyltransferase
