# Supplementary material for: Serum choline is associated with hepatocellular carcinoma survival: a prospective cohort study
Source: Nutr Metab (Lond). 2020 Mar 30;17:25. doi: 10.1186/s12986-020-00445-z (PMC7106561; doi:10.1186/s12986-020-00445-z)
Supplement: Supplementary file 1 — Additional file 1 Table S1. Screening procedure of variables by nonparametric log-rank test. [file 12986_2020_445_MOESM1_ESM.docx]

Supplementary Table 1. Screening procedure of variables by nonparametric log-rank test.

| **Variables** | | ***P*-value for log-rank test** | |
| --- | --- | --- | --- |
| **Sex** | | 0.30 | |
| **Age at diagnosis, years** | | 0.13 | |
| **BMI at diagnosis, kg/m^2^** | | 0.03 | |
| **Education level, n (%)** | | 0.00 | |
| Primary school or below | |  | |
| Secondary & High school | |  | |
| College or above | |  | |
| **Residence, n (%)** | | 0.09 | |
| Urban | |  | |
| Rural | |  | |
| **Smoking status, n (%)** | | 0.02 | |
| Current | |  | |
| former | |  | |
| Never | |  | |
| **Alcohol drinking status, n (%)** | | 0.53 | |
| Current | |  | |
| former | |  | |
| Never | |  | |
| **With family history of PLC, n (%)** | | 0.24 | |
| **HBV or HCV infected, n (%)** | | 0.53 | |
| **AFP≥400 ng/L, n (%)** | | <0.001 | |
| **CRP≥3.0 mg/L, n (%)** | | <0.001 | |
| **Presence of chronic diseases, n (%)** |  | |  |
| Hypertension | | 0.10 | |
| Diabetes mellitus | | 0.13 | |
| Fatty liver | | <0.001 | |
| Cirrhosis | | <0.001 | |
| **Baseline liver damage level, n (%)** | | <0.001 | |
| 1 | |  | |
| 2 | |  | |
| 3 | |  | |
| **Child-Pugh class, n (%)** | | 0.00 | |
| A | |  | |
| B | |  | |
| **BCLC stage, n (%)** | | <0.001 | |
| 0 | |  | |
| A | |  | |
| B | |  | |
| C | |  | |
| **Treatment, n (%)** | | <0.001 | |
| Liver resection | |  | |
| Radiofrequency ablation | |  | |
| Intervention | |  | |
| Others | |  | |
| **Serum choline (μmol/L)** | |  | |
| Median | |  | |
| Interquartile range | |  | |
| **Serum betaine (μmol/L)** | |  | |
| Median | |  | |
| Interquartile range | |  | |
| **Serum folate (μmol/L)** | | 0.09 | |
| Median | |  | |
| Interquartile range | |  | |
