# Supplementary material for: Serum choline is associated with hepatocellular carcinoma survival: a prospective cohort study
Source: Nutr Metab (Lond). 2020 Mar 30;17:25. doi: 10.1186/s12986-020-00445-z (PMC7106561; doi:10.1186/s12986-020-00445-z)
Supplement: Supplementary file 2 — Additional file 2 Table S2. Baseline characteristics of female and male HCC patients. [file 12986_2020_445_MOESM2_ESM.docx]

Supplementary Table 2. Baseline characteristics of female and male HCC patients.

| **Variables** | **Total cases (n=866)** | **Women (n=96)** | **Men (n=767)** |
| --- | --- | --- | --- |
| **Age at diagnosis ^a^, years** | 52.3±11.8 | 51.6±14.0 | 52.3±11.5 |
| **BMI at diagnosis ^a^, kg/m^2^** | 22.7±3.3 | 22.6±3.8 | 22.7±3.2 |
| **Education level, n (%)** |  |  |  |
| Primary school or below | 171 (19.8) | 40 (41.7) | 131 (17.1) |
| Secondary & High school | 528 (61.2) | 42 (43.8) | 486 (63.4) |
| College or above | 164 (19.0) | 14 (14.6) | 150 (19.6) |
| **Residence, n (%)** |  |  |  |
| Urban | 595 (68.7) | 62 (64.6) | 533 (69.2) |
| Rural | 271 (31.3) | 34 (35.4) | 237 (30.8) |
| **Smoking status, n (%)** |  |  |  |
| Current | 265 (30.6) | 0 (0.0) | 265 (34.4) |
| former | 241 (27.8) | 1 (1.0) | 240 (31.2) |
| Never | 360 (41.6) | 95 (99.0) | 265 (34.4) |
| **Alcohol drinking status, n (%)** |  |  |  |
| Current | 237 (27.4) | 3 (3.1) | 234 (30.4) |
| former | 124 (14.3) | 0 (0.0) | 124 (16.1) |
| Never | 505 (58.3) | 93 (96.9) | 412 (53.5) |
| **With family history of PLC, n (%)** | 112 (12.9) | 13 (13.5) | 99 (12.9) |
| **HBV or HCV infected, n (%)** | 789 (91.1) | 86 (89.6) | 703 (91.3) |
| **AFP≥400 ng/L, n (%)** | 358 (41.4) | 55 (57.3) | 303 (39.5) |
| **CRP≥3.0 mg/L, n (%)** | 436 (50.6) | 38 (39.6) | 398 (52.0) |
| **Presence of chronic diseases, n (%)** | |  |  |
| Hypertension | 111 (12.8) | 15 (15.6) | 96 (12.5) |
| Diabetes mellitus | 69 (8.0) | 8 (8.3) | 61 (7.9) |
| Fatty liver | 143 (16.5) | 15 (15.6) | 128 (16.6) |
| Cirrhosis | 544 (62.8) | 58 (60.4) | 486 (63.1) |
| **Baseline liver damage level, n (%)** |  |  |  |
| 1 | 172 (19.9) | 24 (25.0) | 148 (19.2) |
| 2 | 334 (38.6) | 39 (40.6) | 295 (38.3) |
| 3 | 360 (41.6) | 33 (34.4) | 327 (42.5) |
| **Child-Pugh class, n (%)** |  |  |  |
| A | 851 (98.3) | 94 (97.9) | 757 (98.3) |
| B | 15 (1.7) | 2 (2.1) | 13 (1.7) |
| **BCLC stage, n (%)** |  |  |  |
| 0 | 82 (9.5) | 12 (12.5) | 70 (9.1) |
| A | 266 (30.7) | 31 (32.3) | 235 (30.5) |
| B | 95 (11.0) | 6 (6.2) | 89 (11.6) |
| C | 423 (48.8) | 47 (49.0) | 376 (48.8) |
| **Treatment, n (%)** |  |  |  |
| Liver resection | 383 (44.2) | 43 (44.8) | 340 (44.2) |
| Radiofrequency ablation | 85 (9.8) | 14 (14.6) | 71 (9.2) |
| Intervention | 357 (41.2) | 34 (35.4) | 323 (41.9) |
| Others | 41 (4.7) | 5 (5.2) | 36 (4.7) |
| **Serum choline (μmol/L)** |  |  |  |
| Median | 11.72 | 10.7 | 11.89 |
| Interquartile range | 9.52-14.92 | 8.94-13.67 | 9.59-15.10 |
| **Serum betaine (μmol/L)** |  |  |  |
| Median | 64.48 | 56.48 | 65.47 |
| Interquartile range | 52.44-77.65 | 45.63-72.42 | 53.74-78.18 |
| **Serum folate (μmol/L)** |  |  |  |
| Median | 7.04 | 8.97 | 6.86 |
| Interquartile range | 5.28-9.33 | 6.81-10.99 | 5.09-9.10 |

^a^ values are expressed as mean ± S.D..
